# Temperature dependent local atomic displacements in ammonia intercalated iron selenide superconductor

**DOI:** 10.1038/srep27646

**Published:** 2016-06-09

**Authors:** E. Paris, L. Simonelli, T. Wakita, C. Marini, J.-H. Lee, W. Olszewski, K. Terashima, T. Kakuto, N. Nishimoto, T. Kimura, K. Kudo, T. Kambe, M. Nohara, T. Yokoya, N. L. Saini

**Affiliations:** 1 Dipartimento di Fisica, Sapienza Universitá di Roma, P. le Aldo Moro 2, 00185 Roma, Italy; 2 Center for Life NanoScience@Sapienza, Istituto Italiano di Tecnologia, V. le Regina Elena 291, 00185 Rome, Italy; 3 ALBA Synchrotron Light Facility, Carrer de la Llum 2-26, 08290 Cerdanyola del Vallés, Barcelona, Spain; 4 Research Laboratory for Surface Science, Okayama University, Okayama 700-8530, Japan; 5 Research Centre of New Functional Materials for Energy Production, Storage and Transport, Okayama University, Okayama 700-8530, Japan; 6 Department of Physics, Okayama University, Kita-ku, Okayama 700-8530, Japan; 7 Faculty of Physics, University of Bialystok, 1L K. Ciokowskiego Str., 15-245 Bialystok, Poland

## Abstract

Recently, ammonia-thermal reaction has been used for molecular intercalation in layered FeSe, resulting a new Li_*x*_(NH_3_)_*y*_Fe_2_Se_2_ superconductor with T_*c*_ ~ 45 K. Here, we have used temperature dependent extended x-ray absorption fine structure (EXAFS) to investigate local atomic displacements in single crystals of this new superconductor. Using polarized EXAFS at Fe K-edge we have obtained direct information on the local Fe-Se and Fe-Fe bondlengths and corresponding mean square relative displacements (MSRD). We find that the Se-height in the intercalated system is lower than the one in the binary FeSe, suggesting compressed FeSe_4_ tetrahedron in the title system. Incidentally, there is hardly any effect of the intercalation on the bondlengths characteristics, revealed by the Einstein temperatures, that are similar to those found in the binary FeSe. Therefore, the molecular intercalation induces an effective compression and decouples the FeSe slabs. Furthermore, the results reveal an anomalous change in the atomic correlations across T_*c*_, appearing as a clear decrease in the MSRD, indicating hardening of the local lattice mode. Similar response of the local lattice has been found in other families of superconductors, e.g., A15-type and cuprates superconductors. This observation suggests that local atomic correlations should have some direct correlation with the superconductivity.

Among iron-based layered superconductors, the binary FeSe system with PbO-type structure^1^ is apparently the simplest system without any spacer layers, that may represent the multiband electronic structure of these materials. The superconducting transition temperature of the binary FeSe is ~8 K and it can be increased by anion substitution in the ternary FeSe_1−*x*_Te_*x*_, showing a maximum T_*c*_ of ~15 K. The binary FeSe seems to offer less chemical flexibility than the others because of the lack of any spacers between the active FeSe layers, however, the T_*c*_ of FeSe increases sharply to ~37 K^2^ under the high hydrostatic pressure, a clear hope to manipulate it further. A promising way to increase T_*c*_ is the introduction of chemical strain by intercalation of the FeSe layers. Indeed, the binary FeSe was successfully intercalated by alkaline atoms for matching the chemical pressure to the physical pressure, resulting in the discovery of A_*x*_Fe_2−*y*_Se_2_ (A = K, Cs, Tl) materials with a T_*c*_ of ~32 K^3^,^4^. The alkaline atom intercalated system is interesting due to its peculiar microstructural properties in which the superconducting quantum state is embedded in a Mott insulating state characterized by a large magnetic moment^5^, however, its complexity hardly helps to go beyond.

Recently, ammonia-thermal reaction has permitted a simultaneous intercalation of lithium cations, amide anions and ammonia molecules between the FeSe layers, producing Li_*x*_(NH_3_)_*y*_Fe_2_Se_2_ superconductor with T_*c*_ depending on the thickness of the intercalated spacer layer (maximum T_*c*_ ~ 45 K)^6^,^7^,^8^,^9^,^10^,^11^,^12^,^13^. Unlike A_*x*_Fe_2−*y*_Se_2_ compounds, the new system with molecular spacer layer intercalation is crystallographically homogeneous, however, little is known about its local structure. The physical parameters, which are likely to have major role in the superconducting state, are generally controlled by strain/stress fields due to interlayer interactions. For example, when the binary FeSe is produced as a monolayer grown on SrTiO_3_ the T_*c*_ increases upto ~100 K^14^. This notable experimental result further confirms that local physics and defect chemistry is playing a central role in the superconducting mechanism. Indeed, iron-based superconductors are multiband systems with the electronic properties highly susceptible to any small disorder^15^ affecting the local structural parameters^16^,^17^, e.g., the bond angle and the anion height from the Fe-Fe layer. In this context it is important to understand what is the response of the local structure to the intercalation of the molecular spacer layer and superconductivity of the intercalated system. In this work, we have addressed some of these issues and studied the local structure of ammonia intercalated Li_*x*_(NH_3_)_*y*_ Fe_2_Se_2_ by means of temperature-dependent x-ray absorption spectroscopy. We have exploited polarization dependence of the absorption cross-section to have direct access to the near-neighbour distances in single crystal samples of Li_*x*_(NH_3_)_*y*_ Fe_2_Se_2_ using extended x-ray absorption fine structure (EXAFS) measurements performed at the Fe K-edge.

Here, the focus is on the in-plane polarized x-ray absorption measurements providing direct access to both Fe-Se and Fe-Fe atomic correlations. Figure 1 shows (k^2^-weighted) EXAFS oscillations extracted from the in-plane polarized Fe K-edge x-ray absorption spectra measured at several temperatures. The EXAFS oscillations are visible upto high wave vector (k) above the noise level and show clear evolution as a function of temperature. Apart from a thermal damping, the EXAFS oscillations also reveal apparent structural changes (see, e.g., k-range 8–10 Å^−1^), indicating change in the local structure as a function of temperature. Such changes can be better visualized in the Fourier transform (FT) of EXAFS oscillations, displayed in Fig. 2. The FT magnitudes of EXAFS oscillations provide atomic distribution around the selected atomic site. Therefore, in the Fe K-edge EXAFS the axis origin is placed at the photon-absorbing Fe site while the scatterings with neighbouring atoms appear as peaks in the R-space. The FT are not corrected for the scattering phase-shifts and hence the peaks do not appear at the actual distances from Fe. The double peak structure between 1.5 and 3 Å is due to Fe-Se pair (distance ~2.4 Å) and Fe-Fe pair (distance ~2.7 Å). The peaks appearing at longer distances are due to single scattering contributions of distant shells and multiple scatterings involving different paths, i.e., Fe-Fe (distance ~3.8 Å) and multiple scatterings involving Fe-Se (distance ~4.8 Å), Fe-Fe (distance ~5.3 Å) and Fe-Fe (distance ~5.8 Å). The high quality of EXAFS data can be judged from both EXAFS and FT amplitudes, revealing a systematic temperature evolution.

In order to obtain the local structural parameters we have modeled the EXAFS signal using the equation based on single scattering approximation^18^:


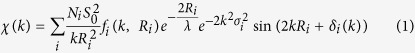


where N_*i*_ is the number of neighbouring atoms at a distance R_*i*_, *δ*_*i*_ is the phase shift, f_*i*_(k, R_*i*_) is the back-scattering amplitude, *λ* is the photoelectron mean free path and 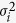 is the EXAFS Debye-Waller factor measuring the mean square relative displacements (MSRD) of the photoabsorber-backscatter pairs. The 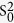 is the so-called passive electrons reduction factor, i.e. EXAFS amplitude reduction factor due to many-body effects related with the losses occurring during the photoelectron propagation in the material (excitations as plasmons, electronhole pairs, etc) and the intrinsic losses due to shake-up and shake-off excitations created by the core-hole in the absorption process. The EXAFS modelling has been carried out by the Artemis package^19^ that uses the FEFF8 code^20^ for the phase and amplitude functions. As a starting structure model we have used tetrahedrally coordinated FeSe taken from the known diffraction results^6^. In the structure, the first coordination shell is composed of 4 Se atoms (at a distance ~2.4 Å) and the second shell is composed of 4 Fe atoms (at a distance ~2.7 Å). The effect of beam polarization (since single crystal samples are used) has been taken into account by introducing an *effective* number of neighbors considering projections of the bond with respect to polarization vector of the x-ray beam. The 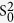 is fixed after the analysis of five different EXAFS scans, providing 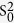 to be 0.5. In the two shells model fits, 5 parameters were floated, i.e., two bond distances (Fe-Se and Fe-Fe), two corresponding *σ*^2^ and the photoelectron energy zero (E_0_). The latter was initially set to 2.5 eV after analyzing different scans at a constant temperature and floated within ±1 eV in subsequent fits to get the best fits at all temperatures. The k-range for the model fits was 3–17 Å^−1^ while the R-range was 1.5–3.0 Å, providing the number of independent data points^21^ 2Δ*k*Δ*R*/*π* to be ~13. Figure 3 shows examples of the two shells model fits (solid lines) in which 5 parameters were allowed to vary.

Figure 4 shows temperature dependence of the local Fe-Se and Fe-Fe bond distances. While the Fe-Se distance tends to increase with temperature (maximum Δ*R* ~ 0.005 Å), the Fe-Fe distance remains basically temperature independent. The anion height from the Fe-Fe layer and the associated bond angle have been proposed to be the key parameters for superconductivity in iron-based materials, having a direct relationship with T_*c*_^16^. The present measurements permit to determine these parameters from the measured bondlengths. Assuming tetrahedral coordination of Se we can determined the Se height (h_*z*_) and Se-Fe-Se bond angle (*α*) using the following relations:


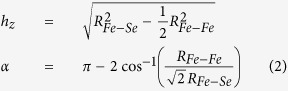


Temperature dependence of h_*z*_ and *α* are shown in Fig. 5. The two parameters are correlated and they basically describe the same effect of the local bondings. Here, the anion height h_*z*_ is ~1.46 Å. Incidentally, the h_*z*_ is found to be ~1.47 Å in FeSe_1−*x*_Te_*x*_ with a negligible doping/temperature dependence^22^,^23^. Therefore, the intercalation of ammonia seems to induce a compression of h_*z*_. Also, the FT peaks (Fig. 2) at longer distances due to Fe-Fe correlations are much stronger in Li_*x*_(NH_3_)_*y*_Fe_2_Se_2_ than in FeSe_1−*x*_Te_*x*_ system^22^,^23^. This is an indication that the Fe-Fe layer in the ammonia intercalated system should be flatter than the one in the ternary FeSe_1−*x*_Te_*x*_. In addition, a small temperature dependent anomaly in h_*z*_ and *α* can be identified, revealing a slight increase in *α* (decrease of h_*z*_) by cooling down across ~200 K and ~50 K. The freezing temperature for ammonia (NH_3_) is ~195 K and it is possible that the change in the chalcogen height/bond angle at ~200 K to be due to a structural change in the intercalating layer, that may be affecting the local correlations of the active FeSe layer. A recent study has reported an anomaly in the quadrupole splitting ~240 K, measured by M*ö*ssbauer spectroscopy, and attributed this to a diffusive motion of Li^+^-ion within the spacer layers^24^. Although, the small anomaly in the present work appears at a lower temperature, we can not rule out the small anomaly to be related with the Li^+^-ion motion, and more work is needed to establish the real cause. On the other hand, the anomaly ~50 K is likely to be related with the superconductivity at which the local Fe-Fe layer is getting thinner and flatter. These small changes can be better seen in the distance-distance correlations given by the *σ*^2^, measuring the MSRD.

The *σ*^2^ is the second moment of the local bondlength distribution function and provides insight to the local bond dynamics. Figure 6 shows temperature dependence of *σ*^2^ for the Fe-Se and Fe-Fe distances, obtained from Fe K-edge EXAFS analysis. The temperature dependence of *σ*^2^(T) can be described by the Einstein model^20^,^25^


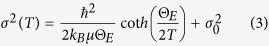


where *k*_*B*_ is the Boltzmann constant, *μ* is the reduced mass of the considered absorber-backscatterer pair and 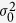 is a offset related with overall configurational disorder. Here, the *σ*^2^(T) for the two bondlengths is well described by the Einstein model down to the transition temperature T_*c*_. However, at *T* < *T*_*c*_ the *σ*^2^ shows a down turn by cooling with a clear deviation from the Einstein-like behavior. Temperature dependent EXAFS permits to determine Einstein temperature Θ_*E*_ describing the bond stiffness. We have used the data between room temperature and ~50 K to determine Θ_*E*_ (see, e.g., model fits shown in Fig. 6). The Θ_*E*_ were found to be 313 ± 10 K and 248 ± 10 K respectively for the Fe-Se and Fe-Fe distances. These values are similar to the known values for the two bonds in the binary FeSe (~318 K and ~268 K^22^,^23^,^26^,^27^,^28^). Thus, local bond correlations in Li_*x*_(NH_3_)_*y*_ Fe_2_Se_2_ are very similar to those in binary FeSe while ammonia intercalation seems to be separating the FeSe layers (increase of h_*z*_), i.e., decoupling without any appreciable change in the local bond characteristics. It is worth mentioning that, while Θ_*E*_ for Fe-Se bonds in K_*x*_Fe_2−*y*_Se_2_ also remains similar to the one in binary FeSe (and hence Li_*x*_(NH_3_)_*y*_ Fe_2_Se_2_), the Fe-Fe bond distance suffers a significant softening due potassium intercalation, with Θ_*E*_ ~ 200 K^26^,^27^.

Another important observation is the abrupt change in the *σ*^2^ at T 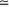 T_*c*_. Such a change has been already observed in the atomic correlations measured in several superconductors^29^,^30^. Here, the change in the *σ*^2^ for Fe-Se bond is much smaller than that for the Fe-Fe bond. Earlier EXAFS studies on iron-based systems have pointed out such a change in the Fe-As correlations^31^, however, it turned out to be very small in the subsequent studies^32^,^33^. The presence of this anomaly, indicating a local mode hardening by cooling across T_*c*_, is a clear evidence of some correlation between electron-lattice interactions and superconductivity. The fact that such a local mode hardening below T_*c*_ has been observed in different superconductors (e.g., cuprates^30^, Nb_3_Ge^29^ and Fe-based materials), it is likely to be common feature of superconductivity phenomena. It should be mentioned that, for the cuprates the anomaly is associated with Cu-O bond correlations. Iron-based pnictides show anomaly to occur in Fe-Fe correlations while the Fe-As hardly showing such an anomaly^34^. This may be related with the fact that the electronic states near the Fermi level in iron-based superconductors are mainly driven by Fe 3d orbitals with a limited contribution from the As p orbitals unlike the case of cuprates in which both Cu 3d and O 2p orbitals are strongly contributing. Incidentally, in the present work the anomaly appears not only in Fe-Fe but also the Fe-Se correlations, likely to be due to different anion chemistry in pnictides and chalcogenides. The fact that the anomaly is not always observed in iron-based systems it can be due to differences in their structural topologies and interlayer interactions depending on the kind of spacer layer. For example, REOFeAsO_1−*x*_F_*x*_ (RE = rare-earth) system contains a well ordered REO spacer layer that may screen the anomaly at T_*c*_ unlike the systems in which the spacer is not well-defined. The BaFe_2_As_2_ is an example of the latter in which the anomalous change at T_*c*_ is well evident^34^. Neverthless, although the anomalous change across T_*c*_ suggests involvement of local lattice in the superconductivity, the question remains if this local lattice is directly causing the superconductivity (acting as a glue) or it is a mere consequence of the superconductivity phenomena, i.e., a transition from the incoherent local atomic correlations (in the normal state) to the coherent atomic correlations (in the superconducting state).

In summary, we have studied the local structure of superconducting Li_*x*_(NH_3_)_*y*_ Fe_2_Se_2_ by means of in-plane polarized Fe K-edge EXAFS measurements performed on single crystal samples. We find that the Se-height from the Fe-Fe layer, determined by the direct measurements of Fe-Se and Fe-Fe bondlengths, appears shrinked in the intercalated system with respect to the binary FeSe. In addition to the compressed FeSe_4_ tetrahedron, the results reveal that the bond length characteristics, measured by the temperature dependence of the mean square relative displacements, hardly show any effect of the molecular layer intercalation. On the other hand, the local structure response to the superconductivity appears as an anomalous change in the MSRD across the superconducting transition temperature T_*c*_, an indication of a sudden change in the atomic correlations in the superconducting phase. It is argued that the higher T_*c*_ of the ammonia intercalated system should be related with an effective compression of the FeSe-layers (with flatter Fe-Fe layer) and interlayer decoupling due to a thick spacer layer. Neveretheless, the results underline importance of the local atomic correlations in the superconductivity of these materials.

## Methods

### Sample synthesis and characterization

Single crystal samples of Li_*x*_(NH_3_)_*y*_ Fe_2_Se_2_ were synthesized using liquid ammonia as a solvent. Several single crystals of FeSe and dopant metal in the appropriate ratio were placed in a glass tube. NH_3_ gas was condensed in the glass tube by cooling with liquid N_2_. The glass tube was filled with liquid NH_3_ (~10 ml) and was sealed. After the intercalation reaction, the liquid NH_3_ was removed by heating and dynamically pumping the glass tube, finally obtaining metal-doped single crystals. The samples were handled in a glove box (O_2_ ≤ 0.5 ppm and H_2_O ≤ 0.5 ppm). The samples were characterized by in-house X-ray diffraction (XRD) from the basal plane of single crystals of parent FeSe and Li_*x*_(NH_3_)_*y*_ Fe_2_Se_2_ using a RIGAKU Ultima IV at room temperature with Co source (*λ* = 1.79 Å). The structural parameters obtained on the single crystal samples studied in the present work are consistent with those reported for polycrystalline samples^6^. The c-axis of the intercalated sample was found to be 17.24 Å for I4/mmm structure. The magnetization measurements were carried out using a SQUID magnetometer (MPMS-R2 and MPMS3, Quantum Design Co. Ltd.). The temperature dependent resistivity was measured using a Physical Properties Measurement System (PPMS, Quantum Design Co. Ltd. with magnetic field up to 9 Tesla). The quantitative composition of the samples was determined by analyzing energy dispersive x-ray (EDX) spectra obtained with a VE-9800SP (Keyence Co. Ltd.) equipped with a scanning electron microscope (SEM). The EDX spectra were obtained from the cleaved surfaces of single crystals of Li_*x*_(NH_3_)_*y*_ Fe_2_Se_2_. Li and NH_3_ concentrations were determined by analyzing inductively coupled plasma (ICP) atomic emission spectroscopy (Vista-pro, Seiko Instruments Co. Ltd.), and found to be x ~ y ~ 0.11 for the samples used in the present study.

### X-ray absorption measurements

Single crystal samples were used for the EXAFS measurements. Temperature dependent x-ray absorption measurements were carried out at the CLÆSS beamline of the ALBA synchrotron in Cerdanyola del Valles (Barcelona) where the synchrotron radiation emitted by a multipole wiggler was monochromatized by a Si(111) double crystal monochromator and Rh-coated mirrors were used to reject higher harmonics. Due to the alkali-metal content, the samples are prone to degradation if placed in ambient atmosphere. To avoid degradation, all the preparation procedures were performed in glovebox under Ar atmosphere, samples were then transfered to the measurement stage. The samples were exfoliated for transmission measurements to reach the desired thickness for absorption jump to be ~1 at the Fe K-edge energy (7112 eV). The absorption experiments were carried out in the normal incidence geometry exploiting three ionization chambers mounted in series to measure the energy-dependent absorption coefficient of the sample and a reference Fe-foil at the same time. In the geometry the polarization of the x-ray beam is parallel to the ab-plane of the single crystal samples. Samples were mounted on a cold finger attached to a Helium cryostat and measured in the temperature range of 13–300 K. The sample temperature was maintained within ±0.5 K during each data acquisition. Several scans were collected for any given temperature to ensure reproducibility and improve the signal-to-noise ratio.

## Additional Information

**How to cite this article**: Paris, E. *et al*. Temperature dependent local atomic displacements in ammonia intercalated iron selenide superconductor. *Sci. Rep.* **6**, 27646; doi: 10.1038/srep27646 (2016).

## Figures and Tables

**Figure 1:**
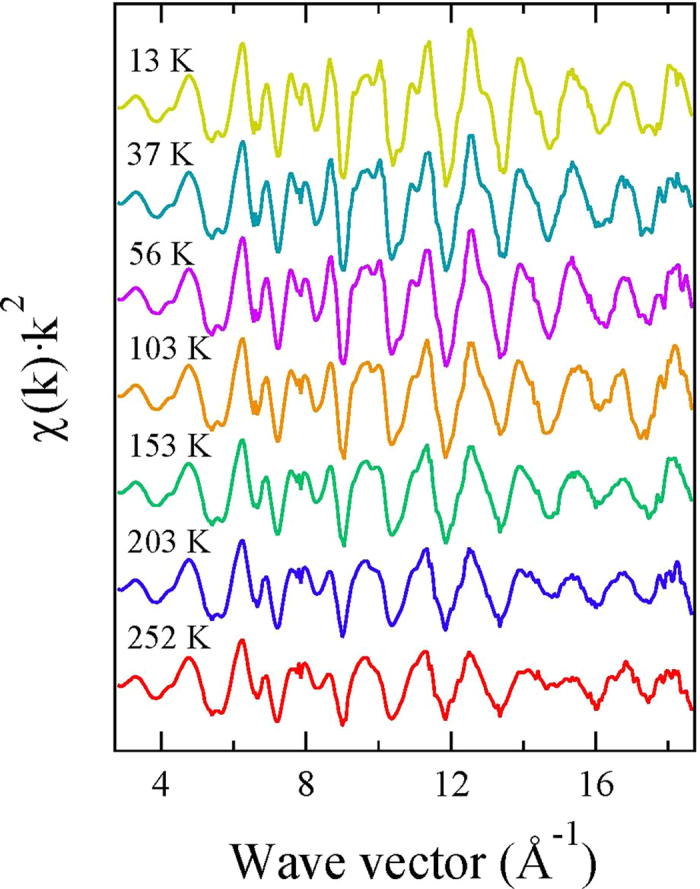
Temperature evolution of the EXAFS oscillations (k^2^-weighted) extracted from in-plane polarized Fe K-edge x-ray absorption spectra measured on single crystal sample of Li_*x*_(NH_3_)_*y*_Fe_2_Se_2_ system. The EXAFS oscillations are shown vertically shifted for a better visualization.

**Figure 2:**
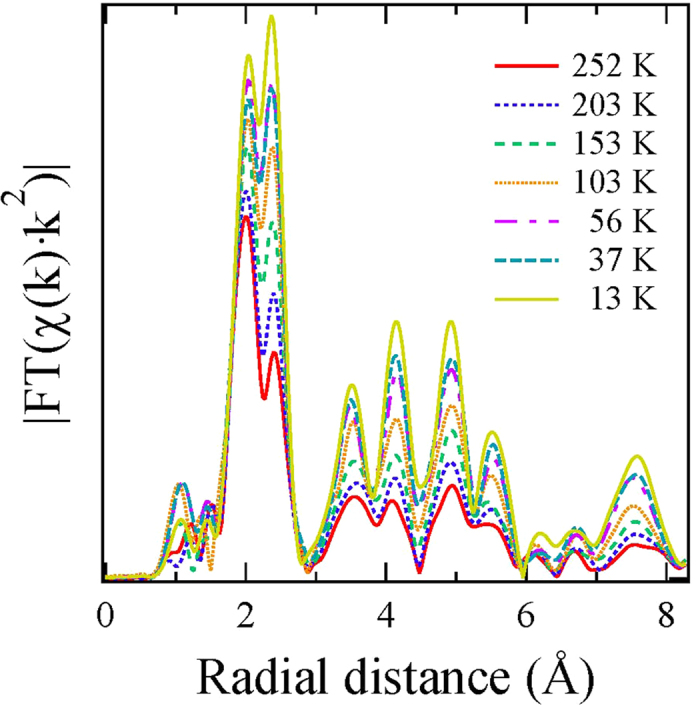
Temperature evolution of Fourier transform (FT) of the EXAFS oscillations measured on Li_*x*_(NH_3_)_*y*_Fe_2_Se_2_ system. The FTs are performed in the k-range 3–17 Å^−1^ using a sine-shaped window.

**Figure 3:**
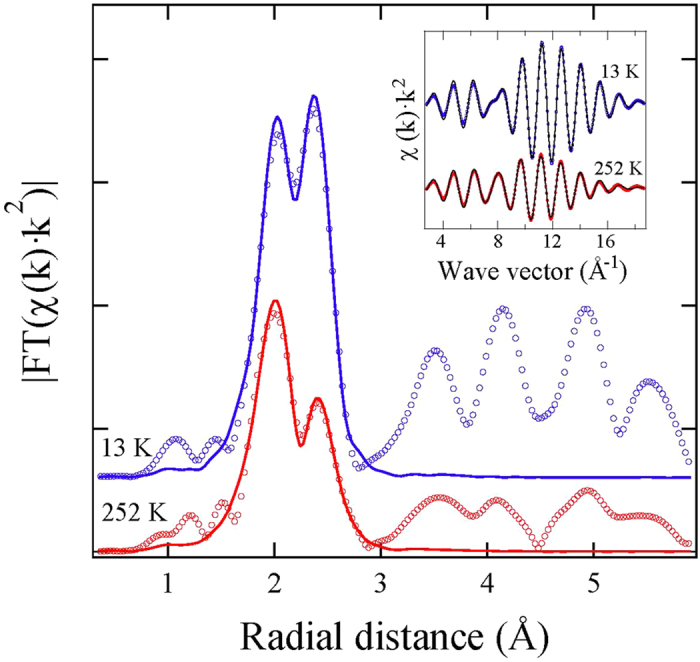
Fourier transforms of *χ*(*k*) · *k*^2^ at two temperatures (empty circles), shown along with the two shells model fits (solid line). The inset shows the respective back Fourier filtered EXAFS oscillations with model fits (R-interval 1.5–3.0 Å).

**Figure 4:**
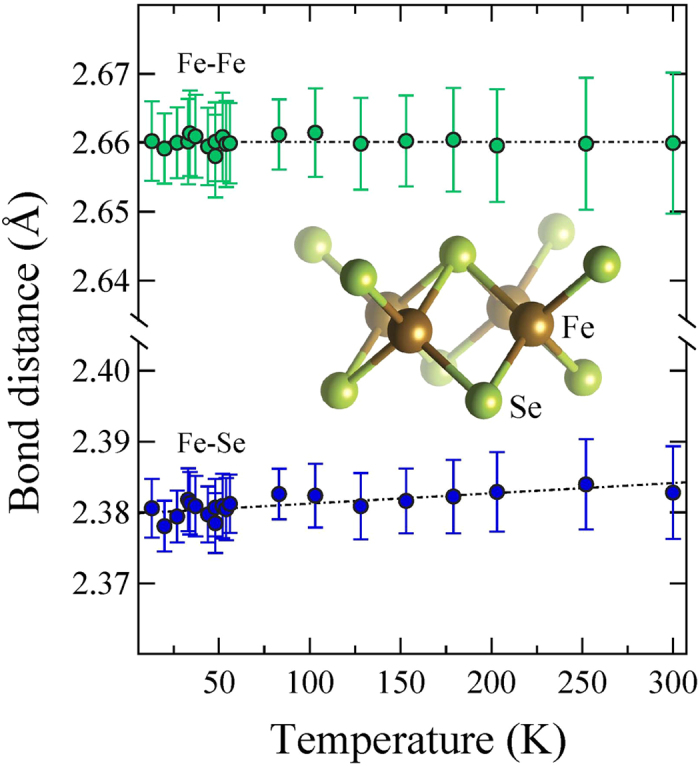
Temperature dependence of the local Fe-Se and Fe-Fe distances obtained from the least-squares fitting of the in-plane polarized Fe K-edge EXAFS oscillations. Inset shows a sketch of the local coordination environment around Fe atom. The dashed line is a guide to the eyes. The error bars represent uncertainties estimated by analyzing different EXAFS scans and considering correlations between different fit parameters.

**Figure 5:**
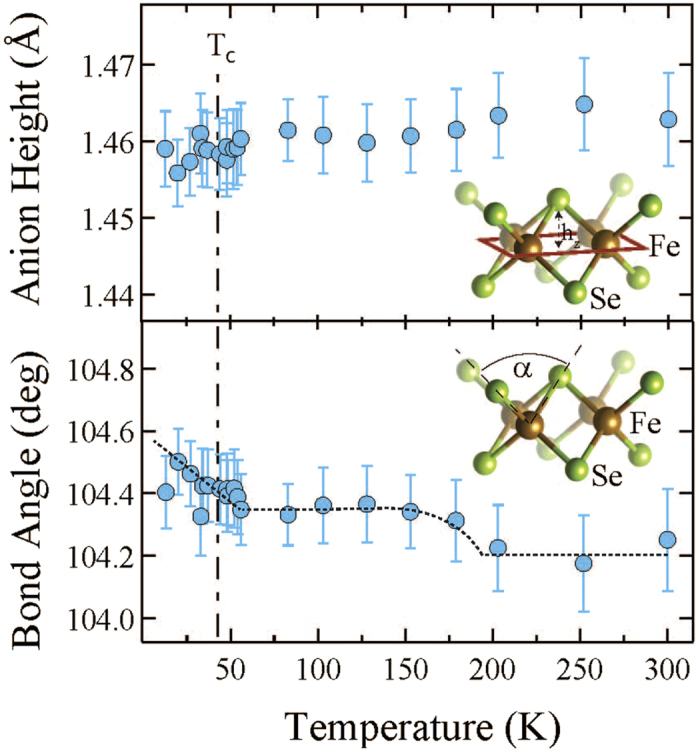
Temperature evolution of the local *h*_*z*_ and *α* parameters i.e. the Se-height from the Fe-Fe layer and the Se-Fe-Se bond angle, respectively. These parameters are determined using the measured bond distances. Error bars take account of the correlations beween the *R*_*Fe*−*Se*_ and *R*_*Fe*−*Fe*_ fit parameters. Cartoons showing definitions of the two parameters are also inserted. The dashed line in the lower panel is a guide to the eyes.

**Figure 6:**
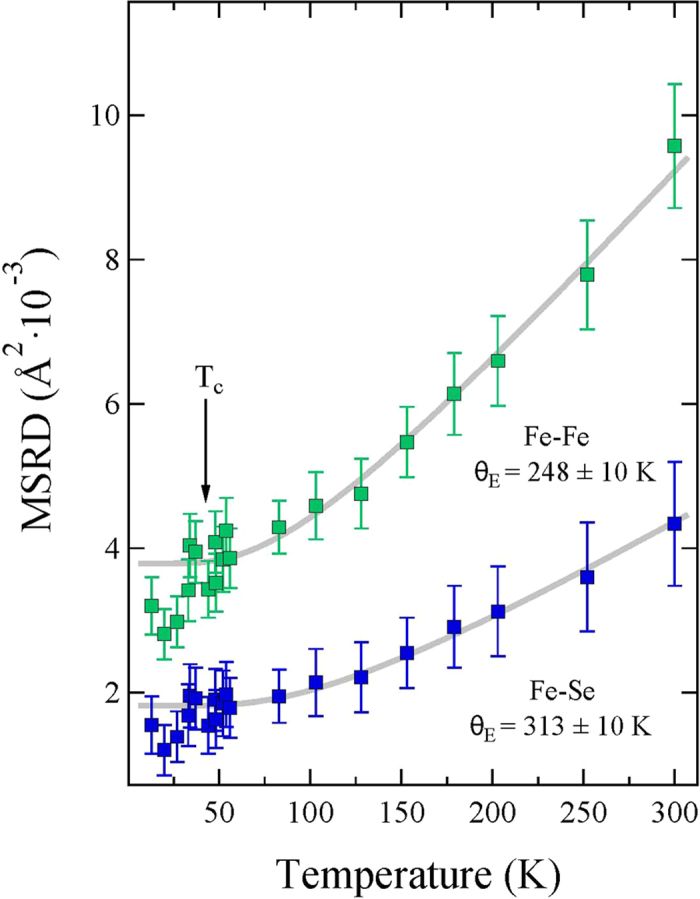
EXAFS Debye -Waller factors describing the mean square relative displacements (MSRD) as a function of temperature for the Fe-Se (blue squares) and Fe-Fe bonds (green squares). The Einstein model (3) fits are shown as grey solid lines.

## References

[b1] Mizuguchi Y. & Takano Y. Review of Fe Chalcogenides as the Simplest Fe-Based Superconductor. J. Phys. Soc. Jpn. 79, 102001 (2010).

[b2] Mizuguchi Y., Tomioka F., Tsuda S., Yamaguchi T. & Takano Y. Superconductivity at 27 K in tetragonal FeSe under high pressure. Appl. Phys. Lett. 93, 152505 (2008).

[b3] Guo J. . Superconductivity in the iron selenide K_*x*_Fe_2_Se_2_ (0 ≤ *x *≤ 1.0). Phys. Rev. B 82, 180520(R) (2010).

[b4] Ying J. J. . Superconductivity and magnetic properties of single crystals of K_0.75_Fe_1.66_Se_2_ and Cs_0.81_Fe_1.61_Se_2_. Phys. Rev. B 83, 212502 (2011).

[b5] Dagotto E. Colloquium: The unexpected properties of alkali metal iron selenide superconductors. Rev. Mod. Phys. 85, 849 (2013).

[b6] Burrard-Lucas M. . Enhancement of the superconducting transition temperature of FeSe by intercalation of a molecular spacer layer. Nat. Mat. 12, 15 (2012). 10.1038/nmat3464 23104153

[b7] Izumi M. . Emergence of double-dome superconductivity in ammoniated metal-doped FeSe. Sci. Rep. 5, 9477 (2015). 25828620 10.1038/srep09477 PMC4381328

[b8] Zheng L. . Emergence of Multiple Superconducting Phases in (NH_3_)_*y*_M_*x*_FeSe (M: Na and Li). Sci. Rep. 5, 12774 (2015). 26239256 10.1038/srep12774 PMC4536530

[b9] Guo J., Lei H., Hayashi F. & Hosono H. Superconductivity and phase instability of NH_3_-free Na-intercalated FeSe_1−*z*_S_*z*_. Nat. Comm. 5, 4756 (2014). 10.1038/ncomms5756 25154371

[b10] Dong X. . Phase Diagram of (Li_1−*x*_Fe_*x*_)OHFeSe: A Bridge between Iron Selenide and Arsenide Superconductors. J. Am. Chem. Soc. 137, 66 (2015). 25532066 10.1021/ja511292f

[b11] Sun H. . Soft Chemical Control of Superconductivity in Lithium Iron Selenide Hydroxides Li_1−*x*_Fe_*x*_(OH)Fe_1−*y*_Se. Inorg. Chem. 54, 1958 (2015). 25613347 10.1021/ic5028702

[b12] Hayashi F., Lei H., Guo J. & Hosono H. Modulation Effect of Interlayer Spacing on the Superconductivity of Electron-Doped FeSe-Based Intercalates. Inorg. Chem. 54, 3346 (2015). 25768303 10.1021/ic503033k

[b13] Yusenko K. V. . Hyper-expanded interlayer separations in superconducting barium intercalates of FeSe. Chem. Commun. 51, 7112 (2015). 10.1039/c5cc01583a 25806691

[b14] Ge J.-F. . Superconductivity above 100 K in single-layer FeSe films on doped SrTiO_3_. Nat. Mat. 14, 285 (2015). 10.1038/nmat4153 25419814

[b15] Mizukami Y. . Disorder-induced topological change of the superconducting gap structure in iron pnictides. Nat. Comm. 5, 5657 (2014). 10.1038/ncomms6657 25430419

[b16] Mizuguchi Y. . Anion height dependence of Tc for the Fe-based superconductor. Supercond. Sci. Technol. 23, 054013 (2010).

[b17] Kuroki K., Usui H., Onari S., Arita R. & Aoki H. Pnictogen height as a possible switch between high-Tc nodeless and low-Tc nodal pairings in the iron-based superconductors. Phys. Rev. B 79, 224511 (2009).

[b18] Bunker G. Introduction to XAFS, Cambridge University Press, 2010; see also *X-ray Absorption: Principles, Applications, Techniques of EXAFS, SEXAFS, XANES*, edited by Prins R. & Koningsberger D. C. (Wiley, New York, 1988).

[b19] Ravel B. & Newville M. ATHENA, ARTEMIS, HEPHAESTUS: data analysis for X-ray absorption spectroscopy using IFEFFIT. J. Synch. Rad. 12, 537 (2005). 10.1107/S0909049505012719 15968136

[b20] Rehr J. J. & Albers R. C. Theoretical approaches to x-ray absorption fine structure. Rev. Mod. Phys. 72, 621 (2000).

[b21] Lee P. A., Citrin P. H., Eisenberger P. & Kincaid B. M. Extended x-ray absorption fine structure - its strengths and limitations as a structural tool. Rev. Mod. Phys. 53, 769 (1981).

[b22] Joseph B. . Evidence of local structural inhomogeneity in FeSe_1−*x*_Te_*x*_ from extended x-ray absorption fine structure. Phys. Rev. B 82, 020502 (2010).

[b23] Iadecola A. . Random alloy-like local structure of Fe(Se, S)_1−*x*_Te_*x*_ superconductors revealed by extended x-ray absorption fine structure. J. Phys. Condens. Matter 23, 425701 (2011). 21983016 10.1088/0953-8984/23/42/425701

[b24] Shylin S. I. . Intercalation effect on hyperfine parameters of Fe in FeSe superconductor with Tc = 42 K. EPL 109, 67004 (2015).

[b25] Sevillano E., Meuth H. & Rehr J. J. Extended x-ray absorption fine structure Debye-Waller factors. I. Monatomic crystals. Phys. Rev. B 20, 4908 (1979).

[b26] Iadecola A. . Large local disorder in superconducting K_0.8_Fe_1.6_Se_2_ studied by extended x-ray absorption fine structure. J. Phys. Condens. Matter 24, 115701 (2012). 22353735 10.1088/0953-8984/24/11/115701

[b27] Iadecola A. . Local structure response of phase separation and iron-vacancy order in K_*x*_Fe_2−*y*_Se_2_ superconductor. Phys. Rev. B 90, 174509 (2014).

[b28] Tyson T. A. . Local structure of the superconductor K_0.8_Fe_1.6+*x*_Se_2_: Evidence of large structural disorder. Phys. Rev. B 85, 024504 (2012).

[b29] Saini N. L. . Temperature-dependent local structure in the Nb_3_Ge superconductor studied by high-resolution Ge K-edge EXAFS measurements. Phys. Rev. B 68, 104507 (2003).

[b30] Saini N. L., Bianconi A. & Oyanagi H. Evidence for Critical Lattice Fluctuations in the High Tc Cuprates. J. Phys. Soc. Jpn. 70, 2092 (2001).

[b31] Zhang C. J., Oyanagi H., Sun Z. H., Kamihara Y. & Hosono H. Electronic and lattice structures in SmFeAsO_1−*x*_F_*x*_ probed by x-ray absorption spectroscopy. Phys. Rev. B 81, 094516 (2010).

[b32] Joseph B., Iadecola A., Malavasi L. & Saini N. L. Temperature-dependent local structure of NdFeAsO_1−*x*_F_*x*_ system using arsenic K-edge extended x-ray absorption fine structure. J. Phys.: Condens. Matter 23, 265701 (2011). 21666306 10.1088/0953-8984/23/26/265701

[b33] Joseph B. . Temperature dependent local atomic displacements in Ru substituted SmFe_1−*x*_Ru_*x*_AsO_0.85_F_0.15_ superconductors. Supercond. Sci. Technol. 26, 065005 (2013).

[b34] Hacisalihoglu M. Y. . A study of temperature dependent local atomic displacements in a Ba(Fe_1*x*_Co_*x*_)_2_As_2_ superconductor. Phys. Chem. Chem. Phys. 18, 9029 (2016). 26966734 10.1039/c5cp07985c

